# Supplementary material for: Host CD59 Potentiates the Type III Secretion System in Yersinia pseudotuberculosis
Source: bioRxiv. 2026 Jun 30:2026.01.07.697569. Originally published 2026 Jan 7. Preprint. [Version 3] doi: 10.64898/2026.01.07.697569 (PMC12803202; doi:10.64898/2026.01.07.697569)
Supplement: Supplementary file 1 [file NIHPP2026.01.07.697569v3-supplement-1.pdf]

Supplemental Figure Legends.

Figure S1. CD59-KO show a kinetic defect in pore formation. Noted cell lines adherent to 96-well dishes were challenged with either the YPIIIΔ5 strain (pore-forming competent) or YPIIIΔB (TTSS-defective) in the presence of propidium iodide (PI) in triplicate wells, and mean PI fluorescence was assayed over time using a multiwell fluorometer at 37°C. Shown are mean ± SE for the 3 determinations.

Figure S2. Pore formation by *Yptb* is T3SS-dependent. CD59KO HEK 293T cells were transfected with a plasmid expressing human CD59, and stable monoclonal cell lines were obtained with CD59 levels that were similar to endogenously expressed protein (WT1 and WT2) or which were overexpressed (OE1 and OE2). **(A)** Indicated cell lines were challenged with YPIIIΔ*yopHMOJE* (YPIIIΔ5) or YPIIIΔ*yopB* (YPIIIΔB) at MOI = 25 for 2 hrs. Cells were labeled with FITC LIVE/DEAD stain and analyzed by flow cytometry to determine extent of pore formation. Data are geometric mean ± SEM of the fluorescence intensity normalized to WT. Data shown is representative of three individual experiments. **(B)** Pore formation peak data showing WT1, WT2, OE1, and OE2 compared to WT and KO after infection by YPIIIΔ5.

Figure S3. Treatment by Phospholipase C removes CD59 from cell surface. WT HEK 293T cells were seeded overnight in 12-well plates. Cells were treated with phospholipase C (PLC)

bioRxiv preprint doi: https://doi.org/10.64898/2026.01.07.697569; this version posted June 30, 2026. The copyright holder for this preprint (which was not certified by peer review) is the author/funder, who has granted bioRxiv a license to display the preprint in perpetuity. It is made available under a CC-BY 4.0 International license.

and incubated at 37°C, 5% CO_2 for 30 mins before harvesting and labeling with FITC-conjugated α-human CD59. Cells were analyzed by flow cytometry for detection of cell surface-localized CD59. Bars note the fluorescence distribution of PLC treated cells.

Figure S4. siRNA knockdown of CD59 is efficient prior to focal adhesion assay. WT HeLa cells were transfected with either siCD59 (blue) or siNT (red) to knock down CD59 expression. Assays were performed after 72 total hours of transfection. Cells were probed with FITC-conjugated α-human CD59 and analyzed by flow cytometry for cell surface localization of CD59. Bars note the fluorescence distribution of siCD59 sample.

Figure S5. Determination of polarity index for perinuclear localization of CtxB-bound GM-1 gangliosides. Polarization indices for theoretical localization patterns as depicted by three cartoons. **A)** Cartoon showing nucleus fully encompassed by CtxB staining: Polarization Index = 0. **B)** Cartoon showing nucleus abutted by CtxB staining with single-sided bias: Polarization Index = 0.97. **C)** Cartoon showing nucleus abutted by CtxB staining showing randomly dispersed puncta: Polarization Index = 0.14. **D)** Examples of regions of interest (ROI), setting DAPI-stained nucleus as the inside limit of ROI, with the outside limit set as 1μm dilation of the displayed nuclei circumferences. **E)** Six individual cells from the dataset generated in Fig. 5, showing examples of circumferential and polarized CtxB staining. Displayed are the calculated polarization indices (R) for the two states.

bioRxiv preprint doi: https://doi.org/10.64898/2026.01.07.697569; this version posted June 30, 2026. The copyright holder for this preprint (which was not certified by peer review) is the author/funder, who has granted bioRxiv a license to display the preprint in perpetuity. It is made available under a CC-BY 4.0 International license.

Figure S6. CD59 knockdown does not change surface levels of GM1 gangliosides. **(A, B)** siNT control cells and siCD59 knockdown cells were probed with FITC-conjugated α-human CD59 **(A)** or immunolabeled for GM1 **(B)** and analyzed by flow cytometry after a 72 hr knockdown.
